# Clinical implications of heterogeneity in PD-L1 immunohistochemical detection in hepatocellular carcinoma: the Blueprint-HCC study

**DOI:** 10.1038/s41416-019-0466-x

**Published:** 2019-05-07

**Authors:** David J. Pinato, Francesco A. Mauri, Paolo Spina, Owen Cain, Abdul Siddique, Robert Goldin, Stephane Victor, Corinna Pizio, Ayse U. Akarca, Renzo L. Boldorini, Luca Mazzucchelli, James R. M. Black, Shishir Shetty, Teresa Marafioti, Rohini Sharma

**Affiliations:** 1 Department of Surgery and Cancer, Imperial College London, Hammersmith Hospital, Du Cane Road, London, W120NN UK; 2 Cantonal Institute of Pathology, Via in Selva 24, 6601 Locarno, Switzerland; 3 Department of Health Sciences, Universitá degli Studi del Piemonte Orientale “A. Avogadro”, Via Solaroli 17, Novara, Italy; 4 Institute of Immunology and Immunotherapy, University of Birmingham, Edgbaston, Birmingham, B152TT UK; 5 Department of Histopathology, University College London Hospital, London, UK

**Keywords:** Immunosuppression, Predictive markers

## Abstract

Programmed cell death ligand-1 immunohistochemical detection (PD-L1 IHC) is a putative predictor of response to PD-1/PD-L1-targeted checkpoint inhibitors. However, there is no gold standard assay in hepatocellular carcinoma (HCC). We evaluated 5 PD-L1 IHC assay platforms (E1LN3, 28-8, 22c3, SP263 and SP142) in 100 HCCs reporting PD-L1 expression in malignant (M) and tumour-infiltrating immune cells (TICs) and non-tumorous cirrhotic tissues (NTICs). We found substantial inter-assay heterogeneity in detecting PD-L1 expression in M (*R*^2^ = 0.080–0.921), TICs (Cohen’s  *κ* = 0.175–0.396) and NTICs (*κ* = 0.004–0.505). Such diversity may impact on the reliability and reproducibility of PD-L1 IHC assays as a predictor of response to immune checkpoint inhibitors.

## Background

Immune evasion through up-regulation of programmed cell death-1 (PD-1) pathway is a pivotal mechanism in the progression of hepatocellular carcinoma (HCC), a disease characterised by dismal prognosis and limited treatment options. Therapeutic reversal of immune exhaustion with anti-PD-1/programmed cell death ligand-1 (PD-L1)-targeted therapies induces responses in only 20% of patients with HCC.^1^

PD-L1 expression by immunohistochemistry (IHC) enriches for response to immune checkpoint inhibitors (ICPIs) in selected tumours.^2^ However, its utility in HCC remains controversial. Albeit its expanding clinical use, the predictive role of PD-L1 IHC status is limited by analytical variability, a factor that can be potentially controlled by standardisation of PD-L1 protein detection techniques in clinical samples. Compelling evidence in non-small-cell lung cancer (NSCLC) and melanoma suggests significant inter-assay heterogeneity in comparative studies of PD-L1 IHC tests due to geographical heterogeneity in PD-L1 expression, antibody used and interpretation.^3,4^ While considerable research efforts are underway to harmonise PD-L1 IHC assays in other solid tumours,^5^ the performance of the various PD-L1 IHC assays available is unknown in HCC. As a result, no recommendation can be made for an optimal PD-L1 IHC test in HCC, a tumour where PD-L1 expression predicts for adverse prognosis, but whose predictive role in defining an increased likelihood of response to ICPI remains unclear.^6^ To address this issue, in Blueprint-HCC we performed a quantitative comparison of five antibody clones used for PD-L1 IHC testing in landmark trials of ICPI.

## Methods

**Fig. 1:**
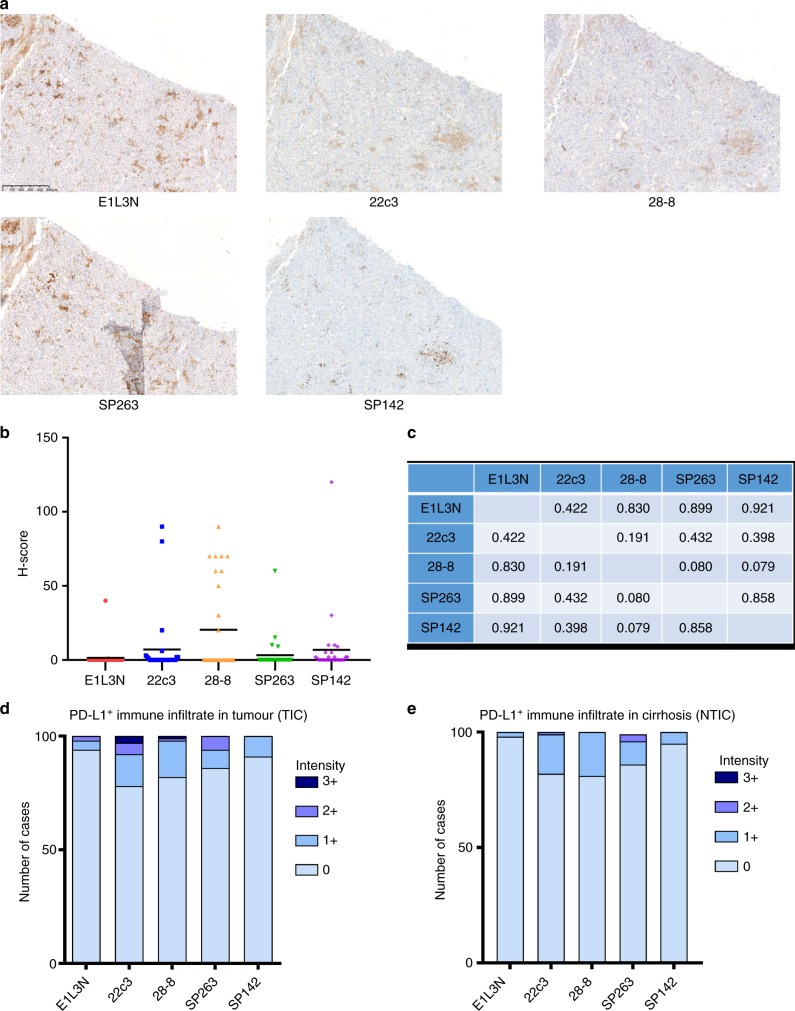
**a** Representative serial tissue microarray (TMA) sections showing patterns of programmed cell death ligand-1 (PD-L1) immunopositivity using E1L3N, 22c3, 28-8, SP263 and SP142 antibodies. Original magnification ×200. **b** Distribution of PD-L1 expression (H-score) in malignant cells across the studied PD-L1 immunohistochemical (IHC) assays in 100 patients with hepatocellular carcinoma (HCC). **c** Pearson’s correlation coefficients for the comparison of H-scores in malignant cells across the studied PD-L1 IHC assays. **d** The proportion of PD-L1-expressing immune cells infiltrating tumour tissue (TIC, *n* = 100). **e** The proportion of PD-L1-expressing immune cells infiltrating the cirrhotic peritumoral tissue (NTIC, *n* = 100)

We constructed tissue microarrays (TMAs) using a multi-centre repository of 100 archival HCC specimens from three tertiary referral centres including Imperial College London (UK, *n* = 41), the academic Liver Unit in Birmingham (UK, *n* = 20) and Novara (Italy, *n* = 39) following ethical approval (Ref. 17/YH/0015). Patients were treated between 2001 and 2016 and clinicopathologic features are presented in Table S1. None of these patients received ICPI therapy. PD-L1 IHC (Fig. S1) with antibody clones E1LN3, 28-8, 22c3, SP263 and SP142 was performed in triplicate cores from tumour (Fig. 1a) and background liver. In tumour cores, PD-L1 expression was evaluated in malignant (M) and in tumour-infiltrating immune cells (TICs). In non-tumorous cores, we reported the presence and intensity of immunopositivity of infiltrating cells (NTICs).

PD-L1 expression was scored in M samples as the percentage of immune-positive cells (1% cut-off) multiplied by chromogenic intensity (ranked from 0 to 3) to derive a semi-quantitative H-score.^7^ For immune infiltrates, PD-L1 positivity was scored semi-quantitatively in a four-tiered system (0–3). Analysis was performed using PRISM (GraphPad, La Jolla, CA, USA). Pearson’s correlation coefficient and Cohen’s *κ* were utilised to evaluate the inter-assay agreement in defining the heterogeneity of PD-L1 expression.^3^

## Results

Tumoral PD-L1 expression was lowest for E1L3N (2%, mean H-score 0.4, standard deviation [SD] 4.0), followed by SP263 (5%, mean H-score 0.9, SD 12.4), 22c3 (9%, mean H-score 2.0, SD 12.1), 28-8 (10%, mean H-score 6.0, SD 18.9) and SP142 (13%, mean H-score 2.0, SD 12.4) (Fig. 1b). In total, 71 cases were PD-L1-negative expression by all tested antibodies. Prevalence of PD-L1-positive TIC was 6% for E1L3N, 22% for 22c3, 18% for 28-8, 14% for SP263 and 13% for SP142. PD-L1-positive NTIC rate was 2% for E1L3N, 18% for 22c3, 19% for 28-8, 13% for SP263 and 5% for SP142.

Despite good staining reproducibility between M triplicates (Table S2), pairwise comparison of H-scores across different antibodies revealed substantial heterogeneity, with highest concordance between E1L3N/SP142 (*R*^2^ = 0.921) and lowest between SP263/28-8 (*R*^2^ = 0.080) (Fig. 1c). Low level of concordance persisted when PD-L1-negative cases were excluded (Table S3). We compared the capacity of each antibody to detect PD-L1-positive immune infiltrates by dichotomising negative cases (score 0) against samples scoring positive for PD-L1 expression at any intensity (scores 1–3). We report significant inter-assay variability in proportion and intensity of PD-L1 expression in immune cell infiltrates (Fig. 1d, e, Fig. S2–3). Cohen’s *κ* testing revealed the greatest level of inter-assay discordance in NTIC versus TIC, suggesting geographical variation as a potential determinant influencing the heterogeneity of PD-L1 expression in HCC (Table 1). No association between PD-L1 positivity and disease characteristics was found (Table S4).

**Table 1:** Inter-assay agreement evaluated by Cohen’s *κ* coefficient in defining the presence of a PD-L1-positive TIC and NTIC in patients with hepatocellular carcinoma (*n* = 100)

	E1L3N	22c3	28-8	SP263	SP142
PD-L1^+^ TIC					
E1L3N	–	0.211	0.175	0.235	0.209
22c3	0.222	–	0.313	0.396	0.222
28-8	0.175	0.313	–	0.257	0.199
SP263	0.235	0.263	0.257	–	0.267
SP142	0.209	0.222	0.199	0.267	–
PD-L1^+^ NTIC					
E1L3N	–	0.038	0.061	0.036	0.030
22c3	0.038	–	0.169	0.505	0.086
28-8	0.061	0.169	–	0.111	0.004
SP263	0.036	0.505	0.	–	0.041
SP142	0.030	0.086	0.004	0.41	–

*TIC* immune infiltrate in tumour, *NTIC* immune infiltrate in cirrhosis

## Discussion

In Blueprint-HCC we document significant inter-assay variability in tumoral and stromal immunolabelling across the principal antibody clones utilised for PD-L1 IHC testing in clinical trials and routine practice. Our observation mirrors the results generated in melanoma and NSCLC, where a number of companion diagnostic assays have evolved in parallel with the clinical development of PD-1/PD-L1-targeting ICPI.^8^ Compared to other tumours, however, the level of inter-assay heterogeneity observed in HCC samples appears even more substantial. Unlike melanoma and NSCLC, HCC is unique for the presence of an immune cell-rich cirrhotic microenvironment, which adds a further layer of complexity to the classification of PD-L1 status. To address spatial and cellular heterogeneity in PD-L1 immunolabelling, we ensured representation of multiple cores from the centre, periphery of HCC and the cirrhotic microenvironment. With this approach, we were able to discover substantial heterogeneity in PD-L1 immunolabelling of immune cell infiltrates, which was maximal for NTIC (Cohen’s *κ* range 0.004–0.505) compared to TIC (0.175–0.396). This is not the first report to suggest the relevance of spatial variance as a determinant of PD-L1 IHC status in malignancy, a finding that might explain the suboptimal linkage between PD-L1 expression and response to PD-1/PD-L1-targeting ICPI. Despite the potential for underestimation in determining PD-L1 status due to the focal nature of protein expression in malignant and immune cells, the use of serial TMA sections might have facilitated a more standardised and less subjective evaluation of cancer specimens as shown in previous studies.^9^ Moreover, in the clinic, the issue of sampling bias is unavoidable as PD-L1 status requires evaluation in biopsy samples, where the quantity of tissue is limited and not dissimilar to that available in a standard TMA section.

Methodologically, the PD-L1 IHC results reported here have been generated using standardised antigen retrieval, immunostaining and scoring techniques, in an attempt to mitigate potential sources of bias. Importantly, there were no qualitative differences in the staining pattern produced by any of the tested antibodies, all of which reproduced the specific staining patterns in the tested samples comparable with those of appropriate positive control reactions.

Based on our findings, the heterogeneity of PD-L1 IHC assays does not appear dependent on the portion of the PD-L1 protein recognised by the tested antibody. When considering immunostaining in M cores, we did not observe significant differences between the antibodies targeting the extracellular (E1LN3, 22c3, 28-8) versus the intracellular domain (SP263, SP142), a finding of clinical importance due to the existence of diverse splicing variants of PD-L1 with uncertain biologic significance, which might have been differentially captured by each antibody.^10^

To conclude, in the Blueprint-HCC study we have shown significant heterogeneity in the performance of PD-L1 IHC assays in HCC. Whether depending on target epitope diversity, differential assay specificity or antibody affinity, our study highlights inter-assay variation to be a critical component of the reliability and reproducibility of PD-L1 expression as a predictive correlate of response to immunotherapy. None of the patients identified in this study have received ICPI treatment for HCC; therefore, we cannot infer a relationship between PD-L1 IHC heterogeneity and response to immunotherapy. Prospective studies evaluating the diverse PD-L1 IHC assays in patient cohorts with adequate linkage with outcomes from PD-1/PD-L1 ICPI treatment are urgently warranted to clarify the clinical meaning of such variation and facilitate patient selection for immunotherapy.

## Supplementary information

Supplementary Results

Supplementary Materials and Methods

## Data Availability

Primary research data are presented in a summative fashion in the manuscript. No publicly available datasets have been generated as part of this work.
