# Supplementary material for: Clinical implications of heterogeneity in PD-L1 immunohistochemical detection in hepatocellular carcinoma: the Blueprint-HCC study
Source: Br J Cancer. 2019 May 7;120(11):1033–6. doi: 10.1038/s41416-019-0466-x (PMC6738063; doi:10.1038/s41416-019-0466-x)
Supplement: Supplementary file 1 — Supplementary Results [file 41416_2019_466_MOESM1_ESM.docx]

**Supplementary Results.**

**Clinical implications of heterogeneity in PD-L1 immuno-histochemical detection in hepatocellular carcinoma: The Blueprint-HCC study.**

David J. Pinato et al.

**Supplementary Table 1.** Clinico-pathologic characteristics of the Blueprint-HCC study cohort.

| **Baseline characteristic** | n=100 |
| --- | --- |
| **Gender**  Male  Female | 73  26 |
| **Age at diagnosis**  Years, median (range) | 68  (33-88) |
| **TNM (7^th^ Edition)**  Stage I  Stage II  Stage III  Stage IV | 48  30  17  5 |
| **Grade**  Well differentiated  Moderately differentiated  Poorly differentiated | 27  54  18 |
| **Microvascular Invasion**  Absent  Present | 67  33 |
| **Number of tumour nodules**  Uninodular  Multinodular | 56  44 |
| **Etiology of chronic liver disease**  Hepatitis C Virus  Alcohol excess  Hepatitis B Virus  Others  Not characterised | 36  35  9  10  10 |
| **Cirrhosis**  Present  Absent | 76  24 |

**Supplementary Table 2**. Intra-class correlation coefficients for the reproducibility of PD-L1 IHC across the three tissue core samples in the entire Blueprint-HCC cohort (n=100).

|  | **Pearson’s R**  **all M cores** | **Number of cases** |
| --- | --- | --- |
| **E1L3N** | 0.99 | N=100 |
| **22c3** | 0.95 | N=98 |
| **28-8** | 0.77 | N=100 |
| **SP263** | 0.96 | N=100 |
| **SP142** | 0.95 | N=96 |

* Too few observations

**Supplementary Table 3**. Pearson’s correlation coefficients for the comparison of H-scores in malignant cells across the studied PD-L1 IHC assays in specimens displaying immunoreactivity for at least 1 PD-L1 IHC assay (n=29).

|  | **E1L3N** | **22c3** | **28-8** | **SP263** | **SP142** |
| --- | --- | --- | --- | --- | --- |
| **E1L3N** | - | 0.359 | 0.223 | 0.528 | 0.351 |
| **22c3** | 0.359 | - | -0.093 | 0.468 | -0.048 |
| **28-8** | 0.223 | -0.93 | - | -.083 | -0.454 |
| **SP263** | 0.528 | 0.468 | -.083 | - | 0.196 |
| **SP142** | 0.351 | -.048 | -0.454 | 0.196 | - |

**Supplementary Table 4**. The relationship between PD-L1 expression in M, TIC and NTIC and salient pathological features of HCC. P-values from Pearson Chi-squrae or Fisher Exact tests are reported for each association as appropriate.

| **Characteristic** | **Malignant Cells (M)** | | | | |
| --- | --- | --- | --- | --- | --- |
|  | **E1L3N^+/-^**  (n=2/98) | **22c3^+/-^**  (n=9/91) | **28-8^+/-^**  (n=10/90) | **SP263^+/-^**  (n=4/96) | **SP142^+/-^**  (n=13/86) |
| **Etiology,**  Viral / Non-viral  (n= 45/55) | 0.20 | 0.72 | 0.75 | 0.34 | 0.37 |
| **Grade,**  1 / 2-3  (n= 27/73) | 0.43 | 0.13 | 0.93 | 0.18 | 0.53 |
| **Tumour Nodules**  Uni / Multinodular  (n= 56/44) | 0.10 | 0.49 | 0.74 | 0.31 | 0.99 |
| **Cirrhosis,**  Yes / No  (n= 76/24) | 0.59 | 0.22 | 0.70 | 0.99 | 0.99 |
|  | **PD-L1^+^ Immune infiltrate in tumour (TIC)** | | | | |
|  | **E1L3N^+/-^**  (n=6/94) | **22c3^+/-^**  (n=22/78) | **28-8^+/-^**  (n=18/82) | **SP263^+/-^**  (n=14/86) | **SP142^+/-^**  (n=9/91) |
| **Etiology,**  Viral / Non-viral  (n= 45/55) | 0.81 | 0.63 | 0.99 | 0.77 | 0.99 |
| **Grade,**  1 / 2-3  (n= 27/73) | 0.92 | 0.99 | 0.85 | 0.06 | 0.76 |
| **Tumour Nodules**  Uni / Multinodular  (n= 56/44) | 0.69 | 0.82 | 0.60 | 0.99 | 0.17 |
| **Cirrhosis,**  Yes / No  (n=76/24) | 0.63 | 0.78 | 0.76 | 0.17 | 0.99 |
|  | **PD-L1^+^ Immune infiltrate in cirrhosis (NTIC)** | | | | |
|  | **E1L3N^+/-^**  (n=2/98) | **22c3^+/-^**  (n=18/72) | **28-8^+/-^**  (n=19/71) | **SP263^+/-^**  (n=13/87) | **SP142^+/-^**  (n=5/95) |
| **Etiology,**  Viral / Non-viral  (n= 45/55) | 0.49 | 0.60 | 0.45 | 0.62 | 0.99 |
| **Grade,**  1 / 2-3  (n= 27/73) | 0.06 | 0.86 | 0.95 | 0.77 | 0.95 |
| **Tumour Nodules**  Uni / Multinodular  (n= 56/44) | 0.50 | 0.29 | 0.44 | 0.50 | 0.65 |
| **Cirrhosis,**  Yes / No  (n=76/24) | 0.43 | 0.55 | 0.99 | 0.95 | 0.33 |

**Supplementary Figure 1.**

**
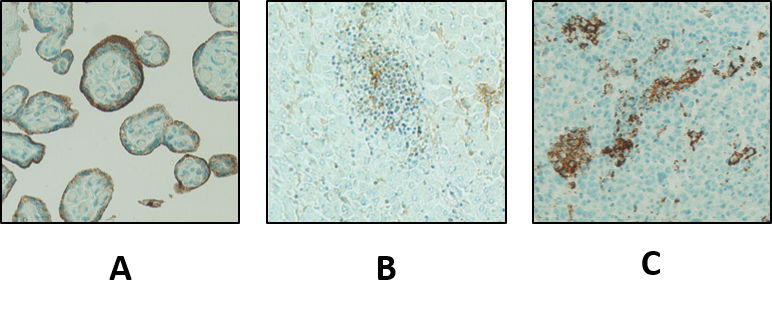
**

Representative sections of tissue microarray sections at 400x magnification showing PD-L1 immunostaining using the SP142 antibody. **A.** Positive internal control (placenta tissue core) showing strong intensity PD-L1 immunopositivity. **B.** A peri-tumoural immune infiltrate showing moderate PD-L1 expression (score 2+). **C.** Focal, high intensity PD-L1 immunostaining in a tumour tissue core (H-score of 120)**.**

**Supplementary Figure 2.**

**
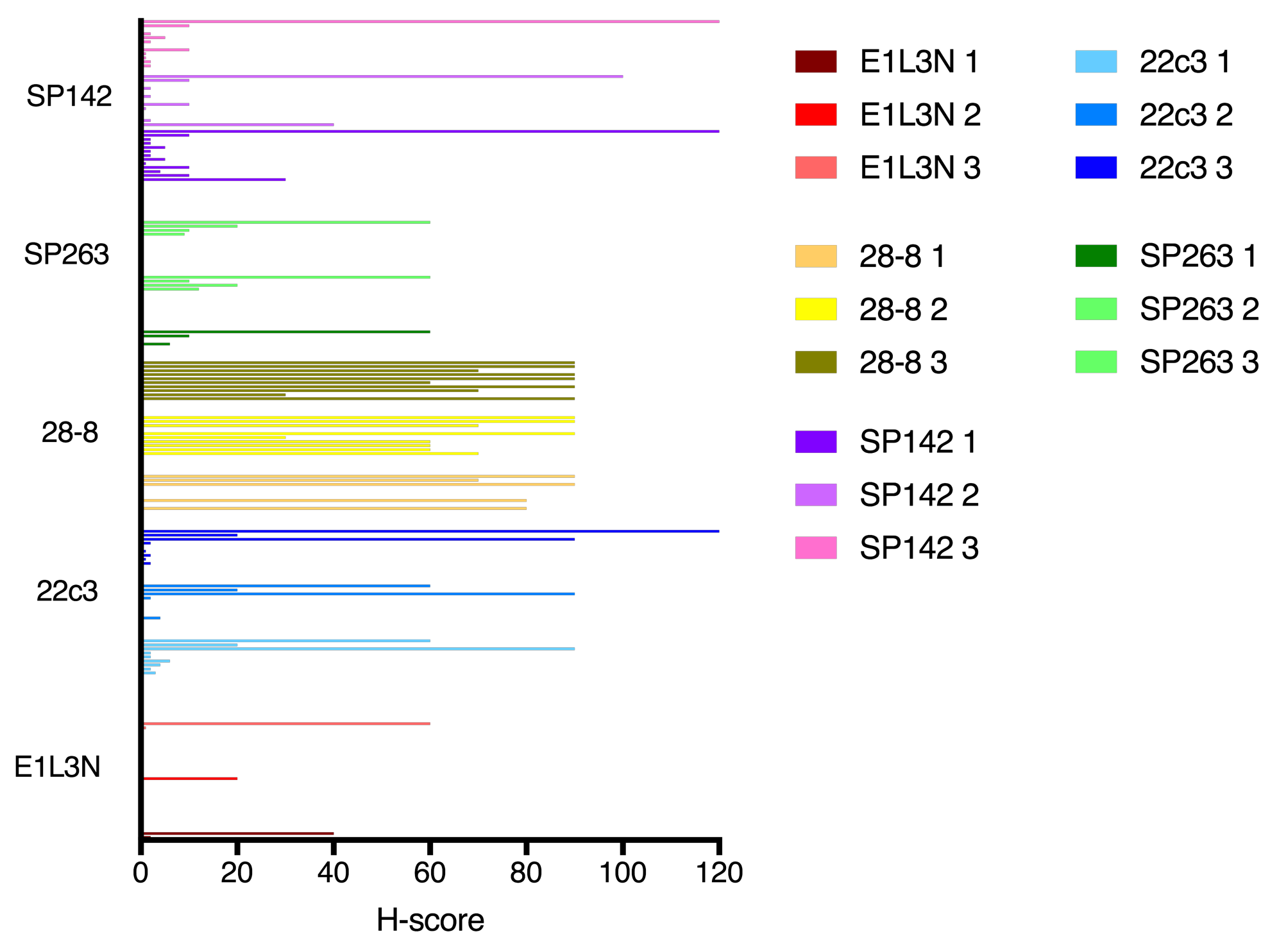
**

Histogram representing the inter-core reproducibility of PD-L1 immunostaining measured by the H-score in triplicate samples of HCC cells across the five tested IHC assays. Patients whose tumour stained positively for at least one PD-L1 IHC assays are displayed (n=29).

**Supplementary Figure 3.**

**
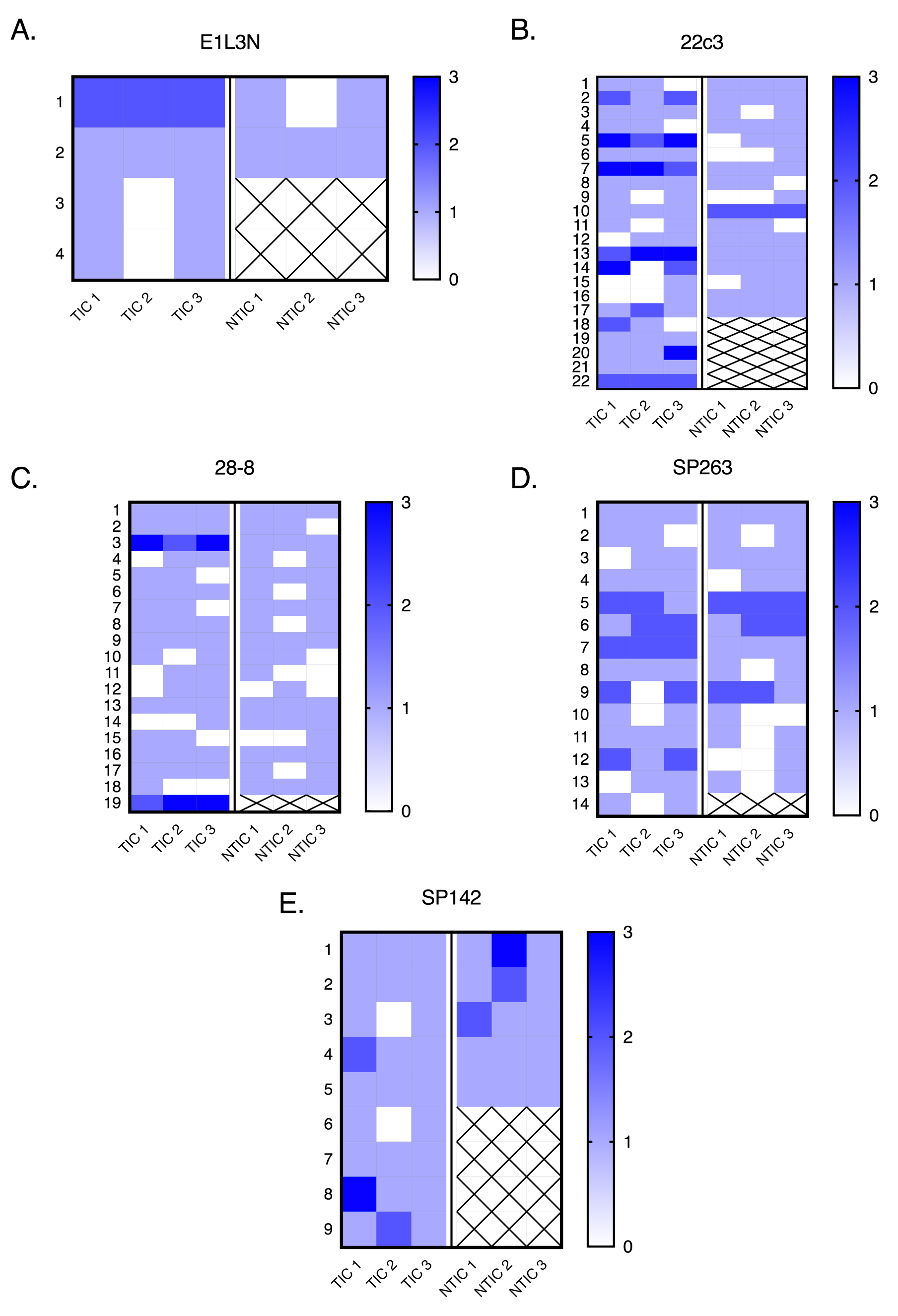
**

Heat-maps representing inter-core variation in PD-L1 labelling of immune cells infiltrating tumour (TIC) and non-tumoural background liver (NTIC) across the 5 studied PD-L1 IHC assays. Intensity of staining was graded on a four-tier system (0-3). Observations reported in each line are independent and not paired for TIC/NTIC.
