# Supplementary material for: Clinical implications of heterogeneity in PD-L1 immunohistochemical detection in hepatocellular carcinoma: the Blueprint-HCC study
Source: Br J Cancer. 2019 May 7;120(11):1033–6. doi: 10.1038/s41416-019-0466-x (PMC6738063; doi:10.1038/s41416-019-0466-x)
Supplement: Supplementary file 2 — Supplementary Materials and Methods [file 41416_2019_466_MOESM2_ESM.docx]

**Supplementary Materials and Methods.**

**Clinical implications of heterogeneity in PD-L1 immuno-histochemical detection in hepatocellular carcinoma: The Blueprint-HCC study.**

David J. Pinato et al.

**Tissue Microarray Construction.**

We generated a collection of archival paraffin-embedded HCC tissues from 3 major academic centres in UK and Italy for a total of 100 patients. A consultant hepatobiliary histopathologist (RG) reviewed all the materials to define key pathologic features (stage, grade) on freshly cut hematoxylin & eosin (H&E) slides. We constructed TMA blocks using an MTA-1 Microarrayer following H&E-slide guided microdissection of target tumour and surrounding non-tumorous areas. We obtained triplicates of 1 mm cores from separate central and peripheral areas of tumor and matching surrounding liver. Adequate sampling of target tissues was confirmed on a freshly cut H&E section from the recipient TMA block before immunohistochemical (IHC) analysis[^1^](#_ENREF_1).

**Immunohistochemistry.**

*E1L3N antibody.*

Immunostaining with the E1L3N antibody (Cat. Nr. 13684 Cell Signaling Technology, Danvers, Massachusetts, USA) was performed on a Leica Bond RX stainer (Leica, Buffalo, Illinois, USA). Antigen retrieval was carried out using a microwave oven at 900W according to standard operating procedures: briefly, the sections were de-paraffinized in xylene, rehydrated in graded alcohols and heated in a microwave oven at 900W for 20 min in citrate buffer at pH 6.0. Tissue slides were incubated in citrate buffer at pH 6.0 for 30 minutes prior to E1L3N immunostaining. Tissue sections were subsequently incubated with the secondary antibody for 1 hour at room temperature and then processed using the Polymer-HRP Kit (BioGenex, San Ramon California, USA) with development in Diaminobenzidine and Mayer’s Haematoxylin counterstaining.

*28-8 antibody.*

The 28-8 antibody was purchased from AbCam (Cat. Nr. ab205921, AbCam, Cambridge, UK) and staining was performed on Leica Bond III automated immunostaining platform with Leica Bond Polymer Refine detection with a DAB chromogen (Leica, DS9800). Peroxide block was performed for 5 minutes, following on-board dewax and epitope retrieval using Leica ER1 solution (Leica, pH6, AR9961) for 20 minutes at 99 ºC. Primary antibody was diluted 1/500 (2ug/ml) in Leica Primary Antibody Diluent (Leica, AR9352) and applied for 40 minutes at room temperature. Antibody detection was performed using a rabbit anti-mouse antibody that was applied for 20 minutes followed by anti-rabbit Polymer-HRP Kit (Biogenex) for a further 20 minutes, both at ambient temperature.

*Dako 22c3, SP263, SP142 antibodies.*

Immunostaining using the Dako 22c3, SP142 and SP263 antibodies was performed using the proprietary immunostaining protocols validated as companion diagnostics for the clinical use of PD-1/PD-L1 targeted inhibitors.

Immunostaining using the Dako 22c3 antibody followed an automated staining protocol validated as part of the PD-L1 IHC 22c3 PharmDx methodology[^2^](#_ENREF_2). Briefly, we used the Dako Autostainer Link 48 platform, where de-paraffinization, rehydration, and antigen retrieval were performed using the PT Link (Dako PT100) using a 3-in-1 procedure. EnVision FLEX target retrieval solution pH 6.1 (Cat. Nr. K8005) was used for antigen retrieval for 20 minutes at 97 ºC. After incubation with the 22c3 primary antibody or species-specific IgG isotype control, TMA sections were incubated with a rabbit anti-mouse linker and subsequently with a ready-to-use visualization reagent consisting of secondary antibody, horseradish peroxidase molecules coupled to a dextran polymer backbone. Development with diaminobenzidine chromogen was subsequently performed and followed by counterstaining with haematoxylin.

PD-L1 immunostaining using the SP263 antibody (Cat. Nr. 790-4905, Ventana Medical Systems, Tucson, AZ, USA) was performed on a fully-integrated Ventana BenchMark ULTRA platform. Following de-paraffinization, tissue slides were baked at 60 ºC for 12 minutes and processed in ULTRA cell conditioning 1 (CC1) solution (Cat. No. 950-224, Ventana) for 64 minutes. After quenching of endogenous peroxidase activity, the primary SP263 antibody or matching isotype control Ig (Cat. No. 790-4795) were incubated at 37 ºC for 32 and 16 minutes respectively. TMA sections were subsequently incubated with OptiView HQ Linker for 8 minutes, followed by incubation with OptiView multimer for another 8 minutes (Cat. No. 760-099, Ventana) and development using the OptiView diaminobenzidine IHC Detection Kit (Cat. No. 760-700, Ventana). Counterstaining was performed with haematoxylin.

PD-L1 immunostaining using the SP142 antibody (Cat. No. 740-4859, Ventana) was also performed with a similar methodology on a Ventana BenchMark ULTRA automatic stainer. Tissue section baking was conducted at 72 ºC for 12 minutes, then processed in CC1 solution for 48 minutes. SP142 primary antibody was incubated for 16 minutes and a rabbit monoclonal Ig (Cat. No. 790-4795) was used as a negative control to assess background staining. Secondary antibody incubation with the OptiView HQ Linker and IHC detection kit followed the methodology described for antibody SP263.

**Biomarker Scoring.**

In tumour cores PD-L1 expression was evaluated in malignant (M) and in tumour-infiltrating immune cells (TIC). Separately, we reported presence and intensity of immunopositivity of infiltrating cells in background non-tumorous cores (NTIC).

In tumour tissue we reported the percentage of immune-positive cells with a 1% cut-off as well as the intensity of the signal (ranked from 0-3) to derive a semi-quantitative histoscore (H-score) as described before[^3^](#_ENREF_3). For immune infiltrates, PD-L1 positivity was scored semi-quantitavely in a four-tiered system (0-3). For all the tested biomarkers, we considered specific tumour cell or peritumoural stromal expression only if membranous or concomitant membranous and cytoplasmic staining were present.

To address the confounding effect of inter-rater reproducibility, two experienced observers (FAM, DJP) scored all the cases independently. After a first assessment, in cases displaying a >20% variation in scoring, disagreement was reconciled by consensus[^4^](#_ENREF_4). A final score was produced by averaging the reads of the 2 scorers and used for statistical analyses.

**Supplementary References.**

1. Pinato DJ, Ramachandran R, Toussi ST, Vergine M, Ngo N, Sharma R, et al. Immunohistochemical markers of the hypoxic response can identify malignancy in phaeochromocytomas and paragangliomas and optimize the detection of tumours with VHL germline mutations. British journal of cancer 2013; 108:429-37.

2. Roach C, Zhang N, Corigliano E, Jansson M, Toland G, Ponto G, et al. Development of a Companion Diagnostic PD-L1 Immunohistochemistry Assay for Pembrolizumab Therapy in Non-Small-cell Lung Cancer. Appl Immunohistochem Mol Morphol 2016; 24:392-7.

3. Pinato DJ, Tan TM, Toussi ST, Ramachandran R, Martin N, Meeran K, et al. An expression signature of the angiogenic response in gastrointestinal neuroendocrine tumours: correlation with tumour phenotype and survival outcomes. British journal of cancer 2014; 110:115-22.

4. Pinato DJ, Black JR, Trousil S, Dina RE, Trivedi P, Mauri FA, et al. Programmed cell death ligands expression in phaeochromocytomas and paragangliomas: Relationship with the hypoxic response, immune evasion and malignant behavior. Oncoimmunology 2017; 6:e1358332.
